# Comparative Analysis of Cytomorphology in Adolescent and Geriatric Patients: A Cross-Sectional Study

**DOI:** 10.7759/cureus.44753

**Published:** 2023-09-05

**Authors:** Neha Kannan, Pratibha Ramani

**Affiliations:** 1 Oral and Maxillofacial Pathology, Saveetha Dental College and Hospital, Saveetha Institute of Medical and Technical Sciences (Deemed to be University), Chennai, IND; 2 Oral Pathology and Microbiology, Saveetha Dental College and Hospital, Saveetha Institute of Medical and Technical Sciences (Deemed to be University), Chennai, IND

**Keywords:** oral epithelium, buccal smear sample, exfoliation, oral exfoliative cytology, aging, cytology, geriatric, adolescent, cytomorphometric analysis

## Abstract

Background

Exfoliation of the surface cells of the normal epithelium occurs as a result of physiological turnover. Epithelial cells are constantly renewed, and they are exfoliated or shed as they migrate from the basal layer to the uppermost layer of the epithelium. Oral exfoliative cytology involves the collection and microscopic evaluation of these shed cells or scraped epithelial cells, quantitatively and qualitatively. The objective of the present study was to analyze and compare the cytomorphometric features like cellular area, nuclear area, and nuclear-cytoplasmic ratio from buccal mucosal smears of adolescent and geriatric patients. This study highlights the changes in cell morphology in different age groups (adolescent and pediatric), which could be attributed to hormonal, habitual, and aging factors.

Material and methods

Buccal smear sample was collected from a total of 60 individuals belonging to the age groups of 11-19 years and above 60 years. The smears were stained with H&E and PAP (Papanicolaou) stain. Photomicrographs were taken in 40x, and measurements were calculated using ImageJ software (National Institutes of Health, Bethesda, Maryland, United States). Cellular size, nuclear size, and nucleo-cytoplasmic ratio were analyzed and compared between the two age groups using independent t-tests using IBM SPSS Statistics for Windows, Version 22.0 (Released 2013; IBM Corp., Armonk, New York, United States).

Results

A significant difference was observed in the cellular area and nuclear area between the two age groups with a p-value of 0.00. No significant findings were present in the nucleo-cytoplasmic area of the two age groups.

Conclusion

Cytomorphometric analysis has shown that there were variations in the cytoplasmic and nuclear areas among different age groups.

## Introduction

As a part of regular physiological turnover, epithelial cells are constantly renewed and they are exfoliated or shed as they migrate from the basal layer to the uppermost layer of the epithelium. The oral epithelium is continuously replaced, indicating that this readily accessible tissue should reveal unambiguous signs of aging. Thus, the oral cavity can be considered as an ideal site to identify the signs of aging [1]. Oral exfoliative cytology involves the collection and microscopic evaluation of these shed cells or scraped epithelial cells, quantitatively and qualitatively. It is a quick, non-invasive, and straightforward approach that has a sensitivity of 89% and a specificity of 89.5% [2].

Exfoliation of the surface cells of the normal epithelium occurs as a result of physiological turnover. The cells in the deeper layer generally adhere to one another. When there is a pathological state, the cells may become less cohesive and they may shed alongside the superficial cells. These exfoliated cells, along with cells that are scraped off using specific tools, can be analyzed numerically or qualitatively to aid in the diagnosis of oral lesions [3].

Numerous debates and conflicts have been sparked by the use of the cytological technique in the oral cavity. Some authors view it as a novel and straightforward diagnostic tool, while others profoundly disagree, believing that it isn't 100% reliable in the field of diagnosing malignancies. With advances, quantitative oral exfoliative cytology has reemerged as a valuable diagnostic technique. In order to establish a diagnosis, a number of variables, including cell size, nuclear size, nuclear-to-cytoplasmic (N/C) ratio, nuclear shape, nuclear membrane discontinuity, optical density, and nuclear texture, can be assessed together at the same time [4]. Morphometry can improve the precision of the cytologic approach in the diagnosis of oral lesions. The capacity to precisely quantify several cell parameters like nuclear diameter, cell diameter, nuclear area, cytoplasmic area, and N/C ratio was enhanced by computer-assisted morphometric examination of cells [5].

The majority of the previous research has been conducted in pathological conditions. There are only limited studies assessing the normal cytomorphology of the cells. The detection of oral premalignant, potentially malignant, or malignant lesions has been accomplished by the use of oral exfoliative cytological techniques. However, the fundamental findings in healthy oral mucosal cells must first be established before the puzzles of pathology can be investigated [2]. Understanding the normal cell morphology is crucial for differentiating it from any abnormal condition. This can serve as a reference scale or basis for comparing pathological smears [6].

The typical oral cytological pattern must be established for both men and women in the context of hormonal fluctuations; with women specifically in relation to the menstrual cycle as the cellular morphology could be highly impacted by sex steroid hormones. Cyclic fluctuations in hormone levels are seen most commonly among adolescent age groups. The maturation of mucosa has been demonstrated to be influenced by abnormal sex hormone levels. It has been observed that well-expressed rhythmic alterations in the oral cavity cells correlate with changes in the hormonal state [7].

Studies have indicated that smoking tobacco affects the cytomorphology of the buccal mucosa. The cellular and immune system response to smoking can lead us to the concept of cytoplasmic or nuclear alterations [8]. According to a study by Ahmed et al., smoking is associated with an increase in nuclear size, N/C ratio, and multilobed nuclei, as well as a decrease in the size of the cytoplasm [9].

Variations in the cytomorphology of the exfoliated cells demonstrate and reflect the numerous events occurring in the body as a result of physiological changes related to aging. The state of the body's local and systemic homeostasis, or its impairment with aging, is often reflected as changes seen in the functional activity of buccal epithelial cells. The discrepancies between the morphological characteristics of buccal nuclei during normal and accelerated aging, according to a few authors, represent the systemic processes of DNA damage, proliferation, and apoptosis of buccal epithelial cells as age advances [10]. These morphological and physical changes have implications for the diagnosis and prognosis of various conditions pertaining to the oral cavity [11].

This study was done to assess the cytomorphometric differences in the adolescent and geriatric age groups. Cellular area, nuclear area, and N/C ratio of the cells from buccal smears were collected and analyzed to estimate the normal baseline values among these age groups, which will further help in the correct diagnosis of pathological conditions.

## Materials and methods

A total of 60 individuals were included in the study after obtaining approval from the Committee on Human Research-Ethical Committee of Saveetha Dental College and Hospitals, Chennai, India (approval number: IHEC/SDC/OPATH-2103/22/662). Simple random sampling was used to choose samples from healthy people who met the inclusion criteria. The participants were divided into two groups: Group 1 comprising the adolescent age group of 11-19 years (n=30) and Group 2 comprising the geriatric age group of > 60 years (n=30). The adolescent participants consisted of 14 males and 16 females while the geriatric age group consisted of 12 males and 18 females. 

**Figure 1:**
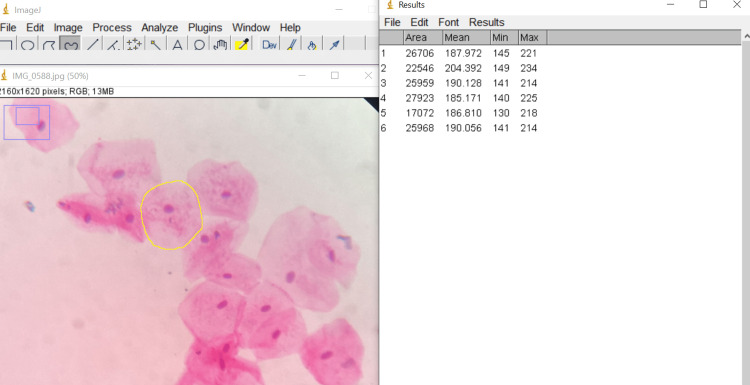
Measuring cellular area of an H&E-stained cell using ImageJ* * Developer: National Institutes of Health, Bethesda, Maryland, United States

**Figure 2:**
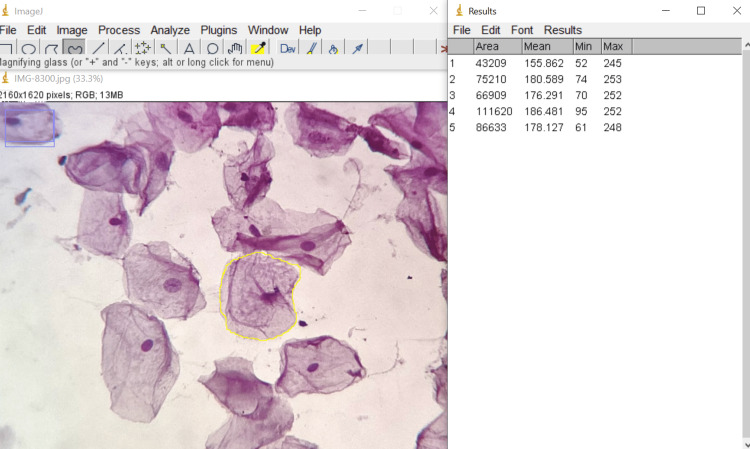
Measuring cellular area of a PAP-stained cell using ImageJ* *Developer: National Institutes of Health, Bethesda, Maryland, United States PAP: Papanicolaou

**Figure 3:**
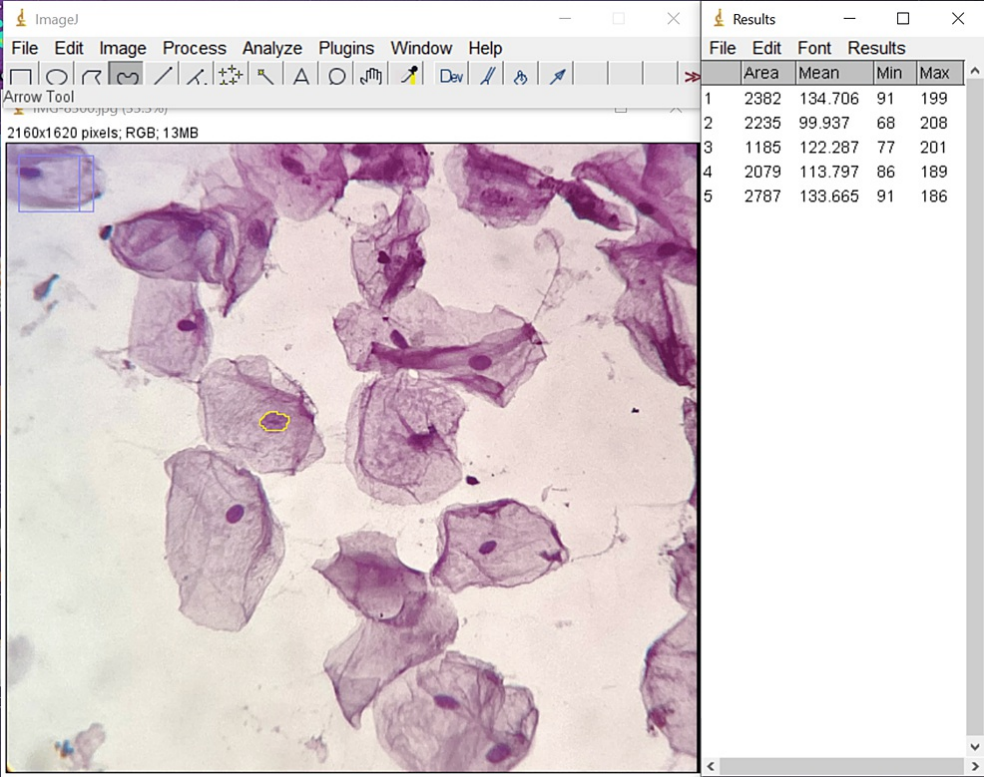
Measuring nuclear area of a PAP-stained cell using ImageJ* * Developed by: National Institutes of Health, Bethesda, Maryland, United States PAP: Papanicolaou

All the patients received a patient information leaflet and were given a detailed explanation of the entire procedure. Each participant gave their informed consent. To remove debris, the patient was instructed to rinse his or her mouth thoroughly with water. Buccal smears were collected using a dampened and sterilized wooden spatula. The smears obtained were spread thinly and evenly in a circular motion on a clean, dry glass slide and fixed using ethanol. The slides were stained using hematoxylin & eosin (H&E) and Papanicolaou (PAP) stains. The stained smears were examined under 40x magnification of an Olympus CH20i microscope (Olympus Corporation, Shinjuku City, Tokyo, Japan). Non-overlapping cell pictures were photographed using iPadOS version 16.05 (Released 2022; Apple Inc., Cupertino, California, United States). The slide was moved in a zigzag motion from left to right to choose the cells. In each case, the mean value was calculated after measuring 15 distinct cells. Following accurate software calibration, all measurements were made using the collected images that were preserved. The parameters assessed were cellular size, nuclear size, and nucleo-cytoplasmic ratio measurements. These were made using ImageJ software (National Institutes of Health, Bethesda, Maryland, United States) for both H&E-stained cells (Figure 1) and PAP-stained cells (Figures 2-3)

For statistical analysis, all measurements were imported from the image analysis software into Microsoft Excel (Microsoft Corporation, Redmond, Washington, United States) using IBM SPSS Statistics for Windows, Version 22.0 (Released 2013; IBM Corp., Armonk, New York, United States). Cellular size, nuclear size, and N/C ratio in relation to age were analyzed using independent t-tests, and graphs were generated. The level of significance was set at p<0.05. Data was reported as means and standard deviation.

Morphometric parameters analyzed

Nuclear Area: A digitalized cursor was used to trace the nucleus' contours in a circle as the software calculated the value simultaneously. The cellular area was also measured similar to how the nuclear diameter was determined. Cytoplasmic area: The difference between the cellular area and the nuclear area was used to calculate the cytoplasmic outline. The N/C ratio was calculated by applying the following formula:

N/C ratio = Nuclear Area/ Cytoplasmic Area

## Results

Mean cellular area

**Figure 4:**
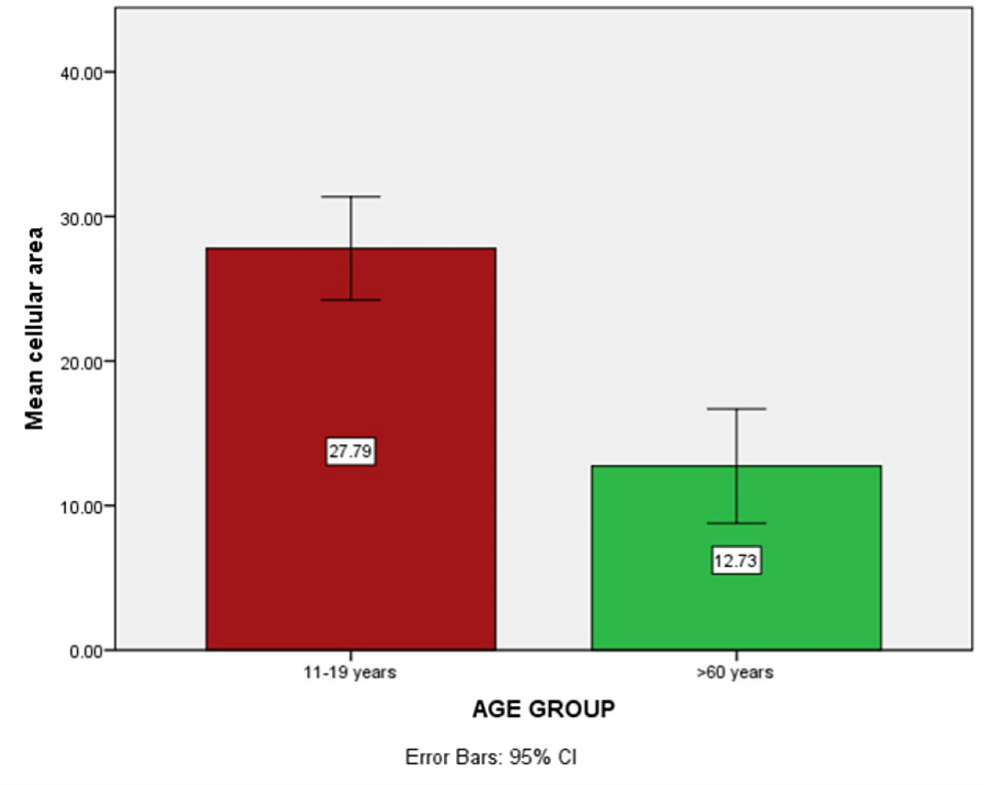
Histogram showing higher mean cellular area in the adolescent age group

The difference in the mean cellular area among the two age groups is shown in Figure 4. The value obtained shows high statistical significance (p value≤0.00) with mean±SD = 27.8± 9.55 in the adolescent age group and 12.73± 10.79 in the geriatric age group. There was a drop in the cellular area of the individuals belonging to the geriatric age group.

Mean nuclear area

**Figure 5:**
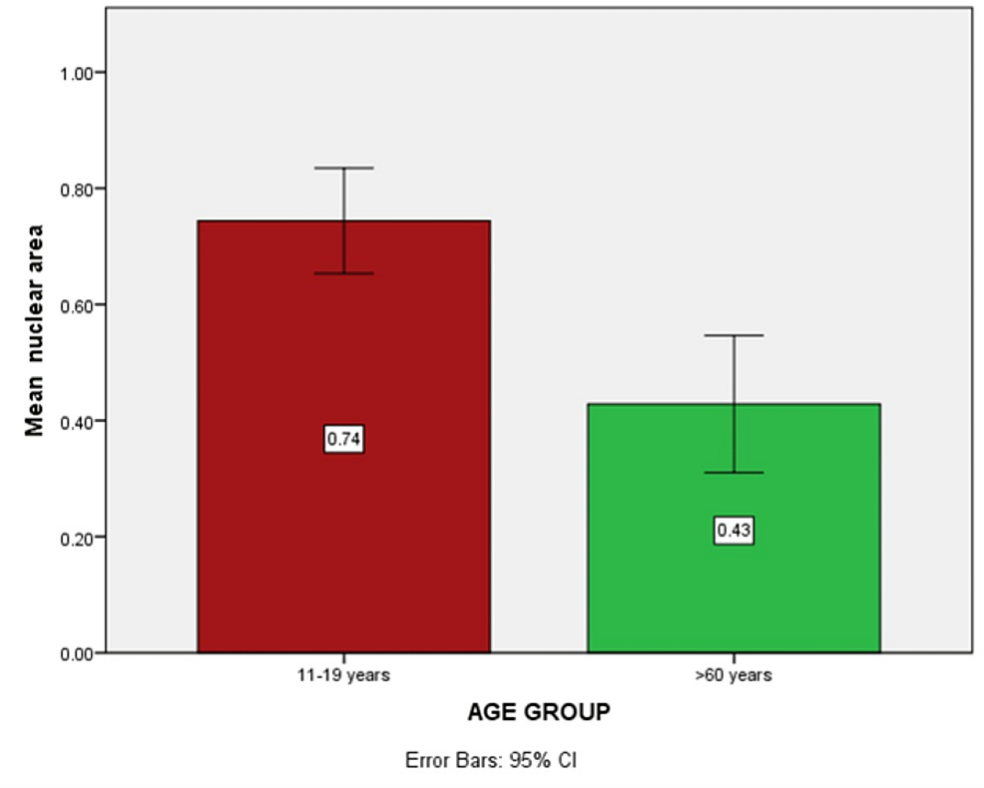
Histogram showing higher mean nuclear area in dolescent age group.

The difference in the mean nuclear area, which is statistically significant among the two age groups, is shown in Figure 5). A p-value of 0.00 was obtained with mean±SD =0.74±0.24 in the adolescent age group and 0.42± 0.31 in the geriatric age group.

Mean N/C ratio

**Figure 6:**
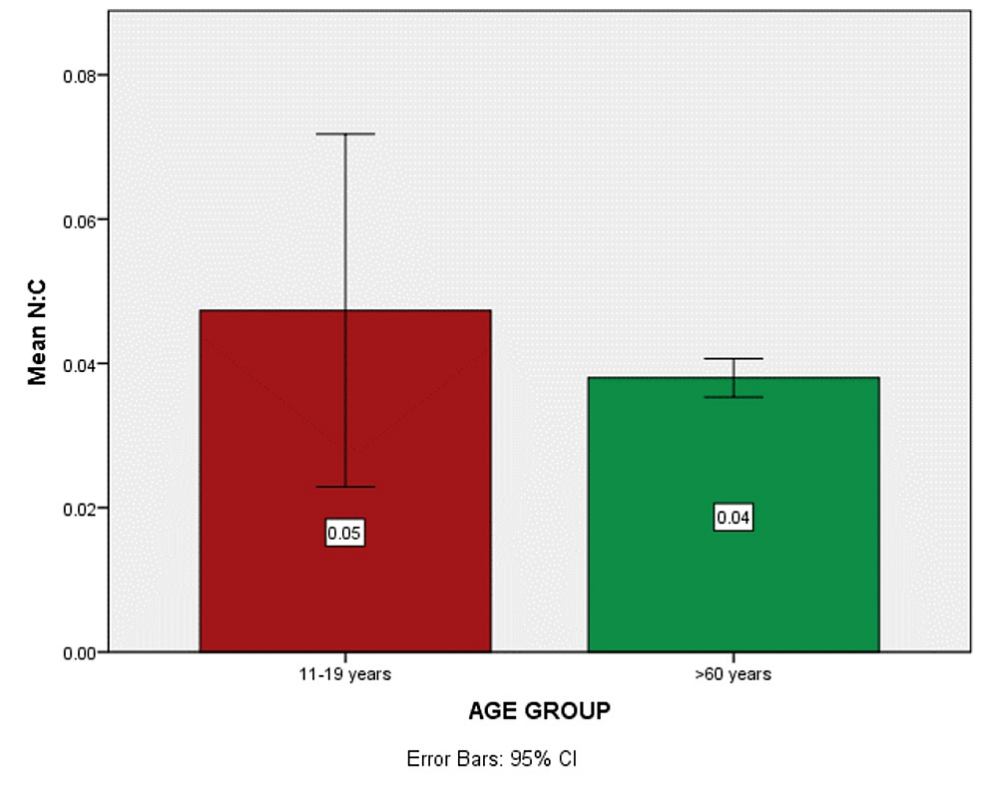
Histogram showing higher nucleo:cytoplasmic ratio in the adolescent age group

The difference in the mean N/C ratio among the two age groups is shown in Figure 6. There is a decline in the N/C ratio as the age advances although the data is not statistically significant (p-value =0.441) with mean±SD =0.047±0.065 in the adolescent age group and 0.038± 0.007 in the geriatric age group.

## Discussion

Cytomorphometric analysis or image analysis of exfoliated cells has been proposed as a key method for defining and identifying cellular as well as nuclear changes as it is faster, more accurate, and reproducible [1].

Various cellular morphological changes have been noted by previous researchers in patients as age advances. Studies comparing cellular alterations have been conducted in the past among different age groups and between genders [12]. However, there is a lack of literature comparing two extreme age groups (adolescent and geriatric) and the factors that could play a role in causing these prominent age-related changes. In the current study, we assessed the variation of cellular area, nuclear area, and N/C ratio among adolescent and geriatric patients with emphasis on the variables bringing about these changes.

In the cellular area, a high statistically significant difference was obtained between the adolescent age group (27.78 mm) and the geriatric age group (12.73 mm) with a p-value= 0.00. High significance was also noted in the nuclear area between age groups (adolescent population 0.74 mm, geriatric population 0.42 mm) with a p-value = 0.00. Changes were also seen in the N/C ratio, but the findings were not statistically significant among the two age groups. These changes could be attributed to hormonal imbalance, habitual factors, various systemic factors, and mucosal atrophy as a result of aging [2,4].

In our study, the nuclear and cellular area along with the N/C ratio was greater in the adolescent age groups when compared to the geriatric patients. These alterations could be caused by the hormonal changes experienced by adolescents, which peak during pubertal development and accelerate cell metabolism and growth, resulting in dimensional changes in the buccal cells. Stratified squamous epithelium experiences cytodifferentiation in response to estrogen. The effects of the estrogen hormone are mediated by cellular receptors. These receptors have been found in a variety of cells throughout the body, including vaginal epithelium [7].

Bercovici et al. compared the hormonal content of menopausal women's vagina and oral mucosa and concluded that maturation values were higher in menopausal women's buccal smears when compared to vaginal smears and that this could be due to local mechanisms or irritating factors rather than hormonal effects [13].

Contradictory to this, Erler et al., in their study, discovered a low level of estrogenic activity in buccal epithelial tissue. Ziskin and Moulton observed that oral cavity cells had sequential changes that were well-expressed and coincided with changes in vaginal smears, representing the hormonal condition of the menstrual cycle [14].

In a study by Laufer et al., estrogen levels gradually increased over the first half of the menstrual cycle before falling. Progesterone levels gradually rose throughout the second half of the menstrual cycle. The levels of the two hormones decrease during menopause. Among the 15-35 years old age group, there was an initial rise in the nuclear and cellular area during the first half of the menstrual cycle followed by a reduction in the second half, but the differences were not statistically significant [15].

These findings are in accordance with our current study, which indicated an initial increase in nuclear diameter and cellular diameter in the adolescent age group followed by a gradual reduction in the size in the geriatric age groups, which could be attributed to the alterations in the hormonal levels during different phases of a woman's menstrual cycle and high peaks of testosterone inducing increased vasculogenesis in adolescent males [7].

Betel leaf chewing and cigarette smoking alter the mechanisms that govern cell development. In a study by Nivia et al., nuclear shrinkage was noted in tobacco users when compared to normal subjects [5]. In another study by Ramaesh et al., individuals who consumed betel leaves and areca nuts and those who were also engaged in smoking had considerably smaller cytoplasmic diameters than those in the control group. This could be attributed to a smoking-dependent cellular adaptation. This adaptative alteration in the cell cytoplasm typically has dysplastic characteristics [16]. Dehydration is a type of cell adaptation in response to the decline in hydration, it could be the cause of the reduction in cytoplasm size seen in the oral mucosa of smokers [9]. Regarding the cytoplasmic area parameter, the aforementioned results were consistent with our research, but they were discordant with reference to the nuclear area. In our study, habit history was not evaluated and its correlation with the cellular changes may reveal valuable information.

Habit-related changes are more common in the older age group as the duration of exposure to the habit is higher when compared to the adolescent age group where the probability for the alteration to commence within a few years of exposure is minimal. The inconsistent findings concerning nuclear size in elderly people may be related to giving up or quitting the habit [5].

Studies in diabetic patients found that the mean cellular area did not show a statistically significant difference (p > 0.001), while the mean nuclear area was significantly greater in the diabetic group (p=0.001) [17]. Because of decreased cell turnover, adenosine triphosphate (ATP) depletion, and a rise in cyclic adenosine monophosphate (cAMP) in diabetic individuals, the cell stays under stress (breakdown product of ATP). When the glycogen-splitting enzyme, phosphorylase, is activated, glucose is released for cell resupply. This compensatory process can be the cause of the change in cellular size [8].

According to the study by Nayar and Sivapathasundharam, cellular area declines with age whereas nuclear area rises. Our findings are consistent with these findings when it comes to cellular area and contradictory in the case of nuclear area [4]. The vast changes between the age groups could be attributed to the increased growth spurt seen in adolescents reflecting on the larger cellular and nuclear sizes, while on the contrary, older age groups show wilting away changes which could be a result of ischemia after atherosclerosis, more common in this age group causing cellular aging leading to a reduction in cellular turnover. As we get older, our cells lose their capacity to function properly due to cell senescence occurring as a consequence of decreased nuclear activity, nuclear deterioration toward the surface, and retarded keratinization [4].

The observations in the present study have revealed that there was an increase in all the parameters (nuclear and cellular area and nuclear-cytoplasmic ratio) among the adolescent age groups when compared to the geriatric age groups. The correlation relating to only one specific feature has been explained by the majority of studies in the literature. This study is an attempt to correlate all the possible perspectives that could be a factor contributing to changes at the cellular level. Cytomorphometric analysis being a non-invasive and rapid technique, can be employed for keeping a lookout for any suspicious lesions in the oral cavity. The values thus obtained can be used as baseline values which will help in differentiating between the normal and abnormal changes in the cellular morphology. The widespread implementation of these techniques, which may increase the diagnostic sensitivity of cytology in the management of oral diseases, is suggested for early results, which are adequately encouraging. 

Limitations

In this study, the total sample size was limited to 60 participants, which is a small sample. Also, a detailed history of the patients related to gender, habits, and systemic diseases could have been collected. 

## Conclusions

Our study results revealed significant age-related variations in cell size, nuclear size, N/C ratio in oral exfoliated cells. It shows the various contributory factors including hormonal changes, habits, systemic, and aging factors, and their impact of insult on cells of the oral cavity. Thus the cytomorphometric data obtained from this study can be used as a benchmark in adolescent and geriatric populations which will aid in improving the sensitivity of the technique and help in distinguishing between the normal and abnormal findings in the oral cavity.
